# Nutritional status, oxidative stress and dementia: the role of selenium in Alzheimer's disease

**DOI:** 10.3389/fnagi.2014.00206

**Published:** 2014-08-28

**Authors:** Jose R. Santos, Auderlan M. Gois, Deise M. F. Mendonça, Marco A. M. Freire

**Affiliations:** ^1^Department of Biology, Federal University of SergipeSão Cristóvão, Brazil; ^2^Department of Bioscience, Federal University of SergipeSão Cristóvão, Brazil; ^3^Laboratory of Cellular Neurobiology, Edmond and Lily Safra International Institute for Neuroscience of NatalNatal, Brazil

**Keywords:** dementia, Alzheimer's disease, nutritional status, selenium, oxidative stress

The term dementia derives from the Latin *demens* (“*de*”: private, “*mens*”: mind, intelligence, judgment—“without a mind”). The American Psychiatric Association (APA) describes it as “any mental impairment, or global cognitive decline in a previously unimpaired person” and is characterized by a deterioration of cognitive, intellectual, emotional, and behavioral skills, severe enough to interfere with the daily life of its sufferers (APA, 1994). It may be elicited by pathologies related to aging, stroke and mechanical injury, or by recurrent use of alcohol and substance abuse, including smoking (DeKosky et al., 2010; Rojas et al., 2010; Brown and Thore, 2011; Rusanen et al., 2011).

Dementias are far more prevalent in the elderly, since only about 5% of all reported cases involve people under 65 years-old, the so-called “early onset dementias” (Fadil et al., 2009). In the United States of America, the prevalence of dementia in individuals aging 90 or over exceeds 37% (Plassman et al., 2007). According to estimates from the Alzheimer's Disease International (ADI), about 36 million people around the world are currently suffering from some sort of dementia and this number was predicted to be at least three times higher in 2050 (ADI, 2010). Approximately two-thirds of all people with dementia live in less developed regions, and these disorders are considered a major burden for health care and social systems in developing countries (Wimo et al., 2003).

Alzheimer's disease (AD) is the major senile dementia, defined as a degenerative, progressive, and irreversible disorder, characterized by a gradual loss of cognitive function and by behavioral disturbances. It most commonly afflicts individuals over 65 years old, accounting for more than half of every worldwide dementia cases (LoGiudice, 2002). AD progression can be ranked into three stages. At the early stage, the patient has difficulty in thinking clearly, presenting a concomitant decrease in performance in complex tasks. At the moderate stage, aphasia is evident—inefficiency in naming objects or to choose the right word to express an idea. In the severe stage, there are prominent changes in the sleep-wake cycle, psychotic symptoms, and in the abilities to walk, talk, and self-care (Herrera-Rivero et al., 2010). Individuals become strictly dependent of caregivers and their condition deteriorates when psychiatric symptoms or often disruptive behavioral changes develop, imposing greater burden to the caregivers. Death usually occurs 3–9 years after the onset of symptoms (Querfurth and LaFerla, 2010).

Currently there is no effective treatment to AD and its cause (or causes) remains to be defined, although factors such as family history, diet, lifestyle, genetics, and head injury have been suggested. Familial AD involves mutations in the amyloid precursor protein and presenilins 1 and 2 (Rogaev et al., 1995; El Kadmiri et al., 2013), which cause overproduction of the β-amyloid protein. Moreover, increased levels of cholesterol, hypertension, and diabetes are also involved in AD development (Bassil and Mollaei, 2012; Wirz et al., 2014). An interdependence between cholesterol metabolism, Apolipoprotein E genotype and β-amyloid metabolic pathway has been reported, with implications to the pathogenesis of AD (Evans et al., 2004). Clinical findings suggest a lower risk of AD in individuals using statins in order to reduce their cholesterol levels (Shepardson et al., 2011).

A common condition in AD patients is weight loss, due to malnourishment induced by poor diet during the progression of the disease and the gradual impotence of feeding appropriately. A healthy nutrition, based on the correct selection and amount of micronutrients, contributes to delaying the cognitive decline both during aging and in AD patients (Lee et al., 2009; Spaccavento et al., 2009; Hadziabdic et al., 2012). Specific foods and diets have been reported to lower the risk of AD (Steele et al., 2007). The Mediterranean diet is based on a dietary pattern of high consumption of plant foods (fruits, vegetables, cereals, and nuts), olive oil as the main source of fat, a moderate intake of fish, and a low consumption of red meat. It is thus rich in omega-3 fatty acids, antioxidants and vitamins, especially B, C, and E (Willett et al., 1995). Antioxidant species are particularly important to help maintain the proper function of the brain (Gomez-Pinilla, 2008; Polidori et al., 2009), and their regular intake reduces oxidative stress (Steele et al., 2007; Otaegui-Arrazola et al., 2014).

Oxidative stress is a major harmful factor during aging and pathological conditions (Finkel and Holbrook, 2000; Freire, 2012). It is involved in the onset and progression of several neurodegenerative disorders, such as multiple sclerosis (Clausen et al., 1988; Gilgun-Sherki et al., 2004), amyotrophic lateral sclerosis (Baillet et al., 2010; Dias et al., 2013), Batten's disease (Clausen et al., 1988), Parkinson's disease (Freire and Santos, 2010; Dias et al., 2013), and AD (Markesbery, 1997; Pratico, 2008; Zhao and Zhao, 2013). Oxidative stress results from an imbalance between the levels of reactive oxygen species (ROS) and endogenous antioxidant mechanisms (Nunomura, 2013; Padurariu et al., 2013), which causes structural and functional impairment of cells by degrading lipids, proteins, and nucleic acids (Reynolds et al., 2007), and ultimately results in cell death.

NADPH oxidase (NOX) is one of the major enzymes involved in the process of oxidative stress. Its overexpression is induced especially by microglial activation in the brain in both acute (Block et al., 2007) and chronic conditions (Wu et al., 2003). NOX seems to play a role in AD, especially by the action of NOX2, which is upregulated in the brain of AD patients (Shimohama et al., 2000; Zekry et al., 2003). NOX2 expression is induced by the presence of β-amyloid plaques that stimulate the activation of microglial NOX leading to superoxide production (Wilkinson et al., 2012), which in turn leads to mitochondrial disfunction (Guimaraes et al., 2009), cleavage of nucleic acids (Nunomura et al., 2012), and proteolysis (Esler and Wolfe, 2001). There is a direct relationship between the impairment of cognitive performance of AD patients and the increase of NOX activity (Ansari and Scheff, 2012).

Glutathione peroxidase (GSH-Px) is a free radical scavenger and a key-enzyme in the endogenous defensive mechanism against free radicals (Chen and Berry, 2003). Its main role is to protect cells from ROS by inactivating hydrogen peroxides and lipid hydroperoxides originated during oxidative metabolism (Arthur, 2000). Accordingly, the decrease of GSH-Px activity leads to tissue damage and cell death due to detrimental action of ROS in increased levels. GSH-Px mechanism of action is based on the redox ability of thiol groups of glutathione and the catalytic reduction of peroxides, either inorganic (hydrogen peroxide) or organic (lipid peroxides). Its functioning is selenium-dependent (Ceballos-Picot et al., 1996), and a low dietary intake of this element alters GSH-Px activity (Arthur, 2000).

Selenium (Se) is micronutrient important to the maintenance of human health, and acts on immune defense, thyroid gland and cardiovascular functions, and cancer prevention (Finley, 2003; Thomson et al., 2009; Joseph and Loscalzo, 2013). Its deficiency results in cardiomyopathy associated to Keshan disease in children (Loscalzo, 2014), a pathology especially observed in regions where the soil is Se-deficient. As stated above, Se acts as an antioxidant component, working in combination with GSH-Px. They protect lipids by catalyzing the reduction of hydrogen peroxide and phospholipid hydroperoxides generated *in vivo* by ROS (Gamble et al., 1997).

Se main natural sources are bread, cereals, seafood, cruciferous vegetables (mainly broccoli) (Finley, 2005; Finley et al., 2005), and especially Brazil nut (Thomson et al., 2008). In an interesting study, Thomson et al. (2008) showed that the insertion of two units of Brazil nuts daily for 12 weeks in the diet increases Se levels in the humans' organism and enhances GSH-Px activity. In an animal model of Parkinson's disease, the systemic administration of Se improved antioxidant activity, protecting dopaminergic cells from the deleterious effects of 6-hydroxydopamine (Zafar et al., 2003), reinforcing the notion of its action in the redox balance in the brain. Se also combines with amino acids to form small peptides called selenoproteins (Papp et al., 2010), which exert antioxidant activities as enzymes (Takemoto et al., 2010; Zhang et al., 2010) and help block ROS involved in cell collapse, as it is seen in AD (Filipcik et al., 2006).

The physiological actions of micronutrients can be enhanced by their association with vitamins, which also play a significant role in reducing oxidative stress in the brain (Morris et al., 1998; Heo et al., 2013; Dysken et al., 2014). In this context, two vitamins are particularly important: vitamin C (ascorbic acid), considered the most important soluble antioxidant, able to neutralize ROS before the initiation of lipid peroxidation (Heo et al., 2013); and vitamin E, an important liposoluble antioxidant that is beneficial particularly at the membrane level, protecting polyunsaturated fatty acids from peroxidation (Tewari et al., 2014). The ingestion of high-dose vitamins E and C supplements may lower the risk of AD (Li et al., 2012; Heo et al., 2013; Dysken et al., 2014).

The clear-cut effects of dietary intake on overall health is well-established (Vermeer et al., 2003), and evidence suggests that nutritional deficiency contributes to the development of neuropathological conditions (Gillman et al., 1995). In a recent meta-analysis, Li et al. (2012) concluded that clinical studies about dietary intake of vitamins E, C, and β-carotene point to a positive role of these elements in prevention and interventional treatment of AD. Regarding Se, studies in humans confirm its participation in the prevention and treatment of brain disorders, either isolated or in combination with other elements (Sanmartin et al., 2011). Smorgon et al. (2004) described a direct correlation between the reduced Se plasma concentration and the decline of cognitive function in AD patients when compared to healthy individuals. Such event can be related to the process of oxidative stress reported in AD. Accordingly, in a recent study Olde Rikkert et al. (2014) found lower levels of Se in the plasma of non-malnourished patients in the early stage of AD when compared to healthy controls, suggesting that differences in nutritional status are present in AD even in the absence of malnutrition (Smorgon et al., 2004; Cardoso et al., 2010; Vural et al., 2010; Olde Rikkert et al., 2014).

Nonetheless, despite the correlations between some nutrients and cognitive function, more research has yet to be realized about the likely wide-ranging impact of nutrition and its benefits to neurodegenerative diseases. Overall, a diet including minerals and vitamins, especially associated to physical activity, importantly contributes to the attenuation of oxidative stress in the brain (Ma, 2008; Morris, 2009; van Praag, 2009). In addition, it is important to remark that the improvement of general nutritional status should be preferably based on food sources instead of alternative supplementation practices, since the formers are more accessible, sustainable, and present lower risk of toxicity than the latters (Finley, 2005).

## Conflict of interest statement

The authors declare that the research was conducted in the absence of any commercial or financial relationships that could be construed as a potential conflict of interest.
